# Associations between sleep trajectories up to 54 months and cognitive school readiness in 4 year old preschool children

**DOI:** 10.3389/fpsyg.2023.1136448

**Published:** 2023-03-28

**Authors:** Elaine Kwang Hsia Tham, Hai-Yan Xu, Xiuju Fu, Rick Siow Mong Goh, Peter D. Gluckman, Yap-Seng Chong, Fabian Yap, Lynette Pei-Chi Shek, Oon Hoe Teoh, Joshua Gooley, Daniel Yam-Thiam Goh, Nora Schneider, Michael J. Meaney, Shirong Cai, Birit F. P. Broekman

**Affiliations:** ^1^Singapore Institute for Clinical Sciences, Agency for Science, Technology and Research (A*STAR), Singapore, Singapore; ^2^Institute of High Performance Computing, Agency for Science, Technology and Research (A*STAR), Singapore, Singapore; ^3^Liggins Institute, The University of Auckland, Auckland, New Zealand; ^4^Department of Obstetrics and Gynaecology, Yong Loo Lin School of Medicine, National University Health System, National University of Singapore, Singapore, Singapore; ^5^Department of Paediatric Endocrinology, KK Women’s and Children’s Hospital, Singapore, Singapore; ^6^Department of Paediatrics, Yong Loo Lin School of Medicine, National University Health System, National University of Singapore, Singapore, Singapore; ^7^Khoo Teck Puat - National University Children’s Medical Institute, National University Health System, Singapore, Singapore; ^8^Respiratory Medicine Service, Department of Paediatrics, KK Women’s and Children’s Hospital, Singapore, Singapore; ^9^Program in Neuroscience and Behavioral Disorders, Duke-NUS Medical School, Singapore, Singapore; ^10^Société des Produits Nestlé SA, Lausanne, Switzerland; ^11^Department of Psychiatry, Faculty of Medicine, McGill University, Montreal, QC, Canada; ^12^Human Potential Translational Research Programme, Yong Loo Lin School of Medicine, National University of Singapore, Singapore, Singapore; ^13^Department of Psychiatry, Amsterdam Public Health Institute, VU University, Amsterdam, Netherlands

**Keywords:** sleep trajectories, school readiness, preschool children, cognitive development, infant sleep

## Abstract

**Purpose:**

This study explores the association between the duration and variation of infant sleep trajectories and subsequent cognitive school readiness at 48–50 months.

**Methods:**

Participants were 288 multi-ethnic children, within the Growing Up in Singapore Towards healthy Outcomes (GUSTO) cohort. Caregiver-reported total, night and day sleep durations were obtained at 3, 6, 9, 12, 18, 24 using the Brief Infant Sleep Questionnaire and 54 months using the Child Sleep Habits Questionnaire. Total, night and day sleep trajectories with varying durations (short, moderate, or long) and variability (consistent or variable; defined by standard errors) were identified. The cognitive school readiness test battery was administered when the children were between 48 and 50 months old. Both unadjusted adjusted analysis of variance models and adjusted analysis of covariance models (for confounders) were performed to assess associations between sleep trajectories and individual school readiness tests in the domains of language, numeracy, general cognition and memory.

**Results:**

In the unadjusted models, children with short variable total sleep trajectories had poorer performance on language tests compared to those with longer and more consistent trajectories. In both unadjusted and adjusted models, children with short variable night sleep trajectories had poorer numeracy knowledge compared to their counterparts with long consistent night sleep trajectories. There were no equivalent associations between sleep trajectories and school readiness performance for tests in the general cognition or memory domains. There were no significant findings for day sleep trajectories.

**Conclusion:**

Findings suggest that individual differences in longitudinal sleep duration patterns from as early as 3 months of age may be associated with language and numeracy aspects of school readiness at 48–50 months of age. This is important, as early school readiness, particularly the domains of language and mathematics, is a key predictor of subsequent academic achievement.

## Introduction

A child’s level of school readiness is defined by multiple factors including its cognitive, socio-emotional, physical development, and temperament (High, 2008). It is important to assess school readiness early as these modifiable measures of how a child is ready to learn can greatly influence subsequent developmental trajectories including social, economic and health outcomes (Williams et al., 2019). In a review of six longitudinal studies (Duncan et al., 2007), researchers found that measures of school readiness (measured in children 4.5 – 10 years of age) in terms of early cognitive (mathematics and language), attention, and socioemotional skills predicted subsequent academic outcomes (at ages 8–14 years), even when adjusting for factors such as sex and socio-economical differences. In particular, early mathematics and language skills were highly predictive of subsequent academic achievement. Attention skills displayed moderate predictive power. Interestingly, socioemotional skills did not predict academic achievement.

There are multiple factors that may influence the level of a child’s school readiness, including family structure (Mistry et al., 2010), parental involvement including home literacy (Williams et al., 2019) and teacher-child communication (Pekdogan and Akgul, 2016). In addition to the above factors, individual differences in (child’s) health-related factors (Currie, 2005) also, directly and indirectly, affect school readiness. Another factor that may be particularly relevant toward school readiness is sleep. Sleep health in young children has been a growing topic of interest. In a recent cross-sectional study amongst 553 Chinese preschoolers (Tso et al., 2016), researchers found that average parent-reported total sleep duration was related to school readiness measured by the Chinese Early Development Instrument (CEDI). The CEDI included five domains, namely, physical health and well-being, social competence, emotional maturity, language and cognitive ability, communicative scales and general knowledge as well as a total CEDI score. Preschoolers who were classified as “sleep deprived” (≤7 h of sleep) had significantly lower total CEDI scores, lower emotional maturity domain scores as well as lower language and cognitive ability domain scores, compared to preschoolers with longer sleep durations. In addition, preschoolers who slept within the 11–12 h recommendation by the National Institute of Health (NIH) had more CEDI domains that were classified as “very ready,” which suggested that these children had greater levels of school readiness than preschoolers who slept below the recommended durations. In another cross-sectional study amongst kindergarten children in Israel, researchers also found that sleep problems were related to school readiness. Kindergarten children who failed to qualify for first grade had shorter sleep durations, greater number of awakenings and longer sleep latencies than their peers who qualified for first grade (Ravid et al., 2009). The above studies suggest that sleep, in particular sleep duration, plays an important role toward measures of school readiness, whereby “poor” sleep, in particular short sleep durations, are associated with poorer levels of school readiness.

The existing findings between sleep duration and school readiness measures draw from cross-sectional point-estimate sleep data, however, it may also be possible that a factor related to poor school readiness may also lead to poor sleep duration. Therefore, it is also important to explore the relation between sleep duration and school readiness through longitudinal group-based sleep duration trajectories because infants and young children may display different types of developmental sleep duration trajectories (Touchette et al., 2007, 2009, 2013; Bruni et al., 2014; Plancoulaine et al., 2018; Smithson et al., 2018; Tham et al., 2021). For example, researchers found that differences in sleep trajectory patterns (short persistent, short increasing, 10- and 11-h persistent) from 2.5 to 6 years of age were related to cognitive performance at school entry. Children with short sleep trajectories had poorer receptive language and non-verbal IQ scores compared to children with longer (10 and 11-h) trajectories (Touchette et al., 2007). However, studies with longitudinal sleep data are scare. To the best of our knowledge, there are no studies exploring how differences in infant sleep trajectories may relate to subsequent school readiness, even though sleep during infancy is known to display greater variance than other stages of life (Iglowstein et al., 2003; Sadeh et al., 2009; Galland et al., 2012).

In a recent study that investigated variations in sleep trajectories across infancy and childhood in Asian children from the Growing Up in Singapore toward Healthy Outcomes (GUSTO) birth cohort, we identified sleep trajectories from early infancy to early childhood (3 months–4.5 years) that varied in duration (short/moderate/long) and variability (variable/consistent) (Tham et al., 2021). Specifically, we identified four patterns of total sleep trajectories (long variable, long consistent, moderate consistent, short variable). The aim of this study is to explore how differences in the above sleep trajectories from 3 months to 4.5 years of age relate to subsequent school readiness performance at 4 years (+2 months) of age.

School readiness is a broad concept and there are no universal tests of school readiness. In addition to school readiness of the individual child, some definitions of school readiness include familial and community support (Williams et al., 2019). Still, most studies acknowledge that school readiness involves key interrelated factors including cognitive, social-emotional and physical development. School readiness in this study of Singaporean preschool children is defined and limited to academic or cognitive school readiness. As mentioned in the earlier paragraphs, cognitive school readiness in preschool children is particularly important for subsequent academic achievement (Duncan et al., 2006).

We hypothesize that children with short night and total sleep trajectories from infancy to early childhood would display poorer performance on these academic/cognitive school readiness test measures compared to their counterparts with longer sleep trajectories. We did not have a directional hypothesis for day sleep to the best of our knowledge, there are no existing studies exploring habitual day sleep and school readiness. Apart from sleep duration, there are also no known studies exploring the relation between variability of sleep trajectories and subsequent school readiness. Therefore, the present study aimed to explore the association between variability of the sleep trajectory patterns and cognitive school readiness test performance in healthy preschool children. We predict that children with consistent sleep trajectory patterns would have better school readiness performance compared to those with variable sleep trajectory patterns.

## Materials and methods

### Participants

Participants in this study were enrolled in the Growing Up in Singapore toward Healthy Outcomes (GUSTO) birth cohort study, which recruited pregnant women aged ≥ 18 years, from two major maternity hospitals in Singapore: KK Women’s and Children’s Hospital and National University Hospital, Singapore. Detailed descriptions of the cohort and methodology used have been reported in previous publications (Soh et al., 2013).

All births occurred between 30 November 2009 and 1 May 2011. For our analyses, we excluded infants from mothers who conceived by *in vitro* fertilization or had a multiple pregnancy and infants who were born preterm (<37 weeks gestational age). At recruitment, there were 55.9% Chinese, 26.1% Malay, and 18% Indian mothers. It should be noted that the general population in Singapore comprises of 74.3% Chinese, 13.4% Malay, 9% Indian, and 3.2% other ethnicities.^1^ Ethnic minorities (Malays and Indians) were oversampled in the GUSTO study so as to improve statistical power in study analyses that take ethnicity subgroups into account. Mean maternal age at recruitment was 30.6 years (range: 18–46 years) and was comparable to the mean maternal age of 31.0 years in the general Singaporean population (see text footnote 1). 3.9% of the women in the GUSTO sample had primary level or below as their highest education attained, 36.0% of the women had Secondary/Technical Education and 60.1% attained GCE A Level/Polytechnic/University degrees. The highest education attained in the general Singaporean population of equivalent age was: 3.9% primary level and below, 16% Secondary/Technical Education, 80.1% GCE A Level/Polytechnic/University degrees (see text footnote 1). Although our cohort may not be representative of the overall Singapore population, the participants were recruited from the two largest maternity hospitals in the country and included both private and subsidized patients.

**FIGURE 1:**
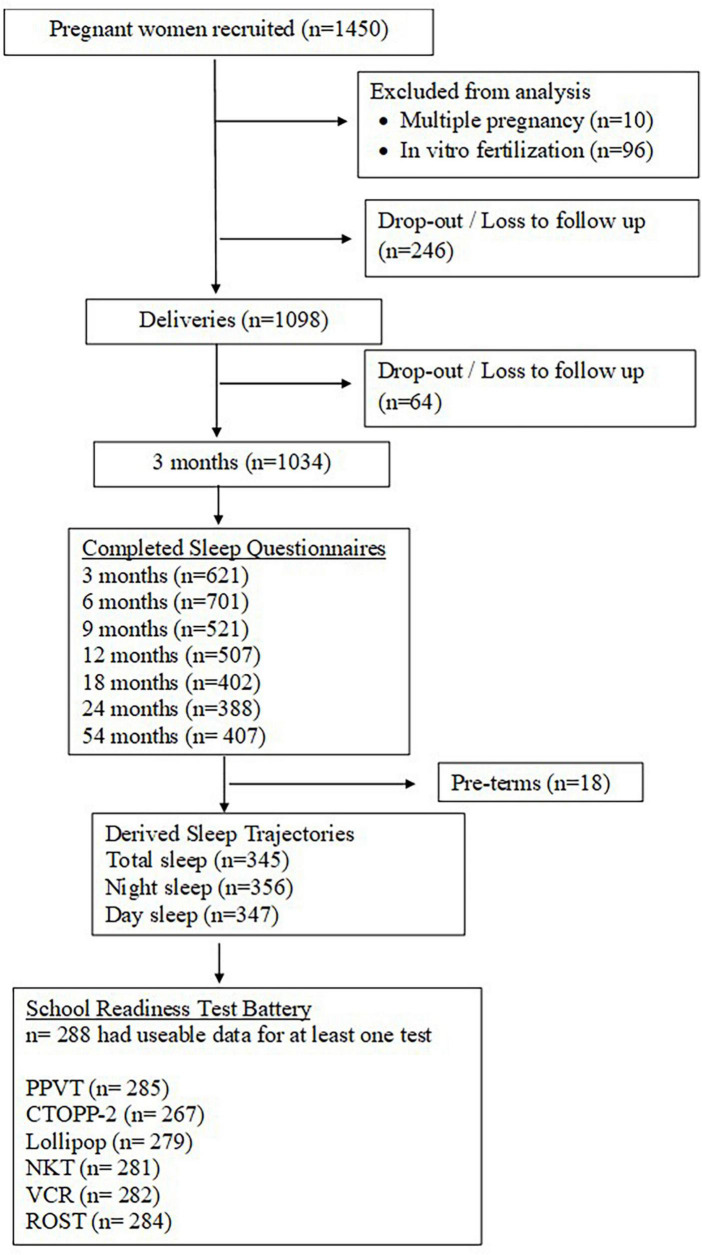
Participant flow diagram. CTOPP-2, Comprehensive Test of Phonological Processing - 2nd edition; NKT, Number Knowledge Test; PPVT-4, Peabody Picture Vocabulary Test - 4th edition; ROST, Random Object Span Test; VCR, Visually Cued Recall.

Out of the 1,450 women recruited, 1,034 mother-infant dyads remained in the study at the 3-month time point (when the first sleep measure was administered). The current study focuses on 288 children, age 3–54 months, with sleep trajectory data based on caregiver-reported sleep duration and who took part in the School Readiness Test battery (see Figure 1).

The GUSTO study was approved by the National Health Care Group Domain Specific Review Board and the SingHealth Centralized Institutional Review Board. Written, informed consent was obtained from each infant’s parent or legal guardian.

### Sleep questionnaires and sleep trajectories

Caregiver-reported information on infants’ sleep duration was obtained from the Brief Infant Sleep Questionnaire (BISQ) (Sadeh, 2004) at 3, 6, 9, 12, 18, and 24 months of age and from the Child Sleep Habits Questionnaire (CSHQ) (Owens et al., 2000) at 54 months of age. For the BISQ, night sleep duration was calculated from caregiver responses to the question [“How much time (on average) does your baby/child spend in sleep during the night?”] and day sleep from the question [“How much time (on average) does your baby/child spend in sleep (naps) during the day?”] over the past 2 weeks. For the CSHQ, night (day) sleep was calculated from responses to the questions “Write in child’s bedtime (usual naptime)” and “Write in the time of day child usually wakes in the morning (after nap).” Total sleep for both BISQ and CSHQ was calculated from the sum of night sleep and day sleep durations.

A Conditional Probabilistic Trajectory Model (CPTM) was used to detect multiple (group-based) sleep trajectories from the sleep data collected. The method and identified trajectories will be briefly described below. Detailed information on the CPTM and sleep trajectories used in this study have been reported in a previous publication (Tham et al., 2021).

**FIGURE 2:**
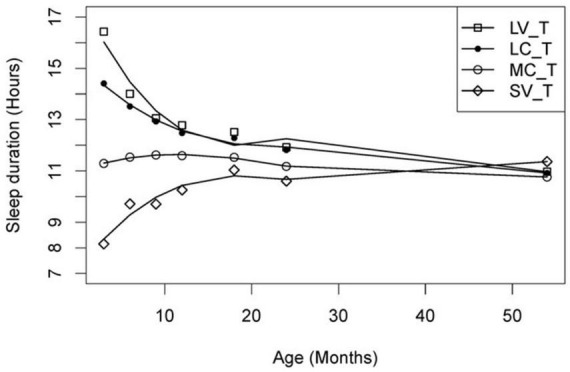
Total sleep duration trajectories. LV_T, long variable total sleep (*n* = 67); LC_T, long consistent total sleep (*n* = 90); MC_T, moderate consistent total sleep (*n* = 97); SV_T, short variable total sleep (*n* = 91). Solid lines depict the model trajectory curve. This figure was published in Tham et al. (2021).

**FIGURE 3:**
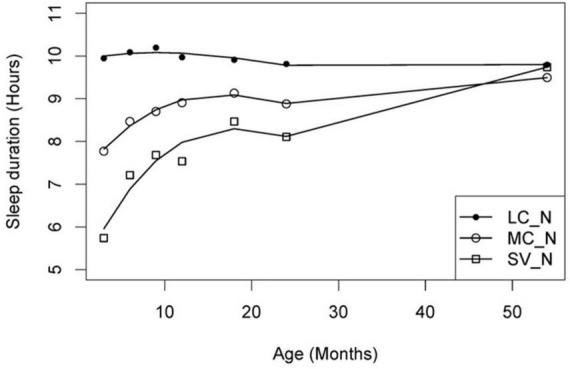
Night sleep duration trajectories. LC_N, long consistent night sleep (*n* = 111); MC_N, moderate consistent night sleep (*n* = 145); SV_N, short variable night sleep (*n* = 100). Solid lines depict the model trajectory curve. This figure was published in Tham et al. (2021).

**FIGURE 4:**
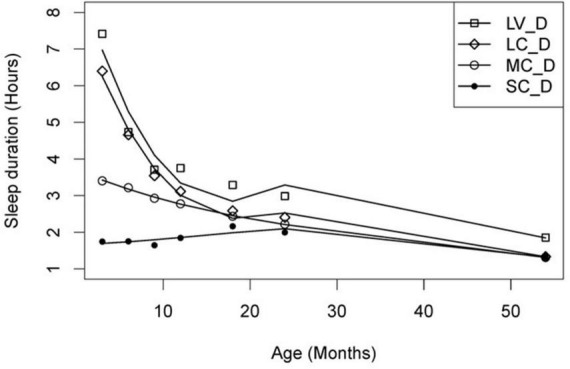
Day sleep duration trajectories. LV_D, long variable day sleep (*n* = 73); LC_D, long decreasing day sleep trajectory (*n* = 71); MC_D, moderate consistent day sleep (*n* = 118); SC_D, short consistent day sleep (*n* = 85). Solid lines depict the model trajectory curve. This figure was published in Tham et al. (2021).

Firstly, individual sleep trajectories were extracted across time using multiple regression models and the children were clustered based on their recorded trajectory parameters. Secondly, a group trajectory was extracted for each group of (clustered) individuals. The children were then reallocated to group-based trajectories by the model based on their maximum likelihood value, i.e., to a group that best represented the individual amongst all the candidate groups. The model’s Bayesian information Criterion (BIC) based on n-fold cross-validation was adopted to determine the number of candidate groups. Based on the above CPTM, group-based sleep trajectories with varying relative durations (short/moderate/long) and variability (consistent/variable) were identified for total sleep (Figure 2), night sleep (Figure 3) and day sleep (Figure 4).

**FIGURE 5:**
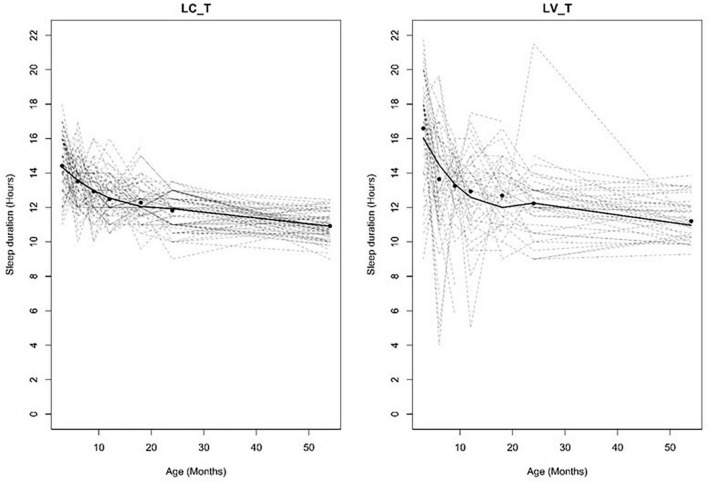
Example of consistent versus variable trajectories. LC_T, long consistent total sleep; LV_T, long variable total sleep. Solid lines depict the model trajectory curve and solid circles depict the study time points. Dotted lines depict individual trajectories. This figure was published in Tham et al. (2021).

Variability was defined by the standard errors in the parameter estimates of the extracted trajectory curves whereby trajectories termed as “consistent” have smaller standard errors than those termed as “variable” (see Figure 5).

The CPTM identified four patterns of total sleep trajectories for 345 children who provided useable sleep data across time, namely long variable (19% of children), long consistent (26%), moderate consistent (28%) and short variable (27%) (Figure 2). Children in the short variable group had significantly shorter sleep durations compared to both long consistent and long variable groups across the 3–24 months of age; and significant shorter sleep duration compared to the moderate consistent group across 3–12 months of age. Children with short variable or long variable trajectories displayed significantly larger between-individual variability (i.e., standard errors) than children with moderate consistent or long consistent trajectories at all study time points (Tham et al., 2021).

Three patterns of night sleep trajectories were derived, namely, long consistent night sleep (*n* = 111), moderate consistent night sleep (*n* = 145) and short variable night sleep (*n* = 100) (Figure 3). Those with long consistent trajectories had approximately 10 h of night sleep throughout the study period. Children with moderate consistent trajectories had around 8–9.5 h of sleep across the study period and children with short variable trajectories had less than 8 h of night sleep from 3 to 12 months, followed by an average of 8 to 9.5 h of sleep from 18 to 54 months of age. In addition, the short variable group had the largest between-individual variability across all timepoints.

For day sleep, four trajectory patterns where identified, namely long variable day sleep (*n* = 73), long consistent day sleep trajectory (*n* = 71), moderate consistent day sleep (*n* = 118) and short consistent day sleep (*n* = 85) (Figure 4). Both long variable and long consistent had a sharp decrease in daytime sleep in the first 9 months, followed by a more gentle decrease from 12 to 54 months of age. The moderate consistent display a gradual decrease in day sleep throughout the study period. In contrast to the long and moderate groups that displayed a decrease in day sleep, children in the short consistent group had a stable amount (approximately 1.5–2 h) of day sleep throughout the study period. In addition, between-individual variability in day sleep duration in the long variable group was significantly larger than all other groups at all timepoints.

It should be noted that existing methods of sleep trajectory modeling (e.g., semiparametric model) assume a constant model variance for all trajectory groups, hence these methods are not able to explore between-individual variations. We chose the Conditional Probabilistic Trajectory Model (CPTM) as it does not assume a constant model variance and hence the CPTM was able to model between-individual variability in sleep and how they differ from the overall developmental patterns of the sample (i.e., the model trajectory curve). For example, children with long consistent sleep display a small variation to the average development sleep duration patterns. In contrast, those with long variable sleep have a larger difference in sleep duration patterns compared to the overall developmental patterns of the sample (Figure 5).

### School readiness test (SRT) battery

A total of 810 children out of 1,069 (participation rate 75.8%) from the GUSTO cohort took part in the school readiness test (SRT) battery when they were between 48 and 50 months old. There were 695 remaining children (with useable data for at least one SRT test) after exclusion criteria were applied. Out of these 695 children with useable data for at least one SRT test, 288 had sleep trajectory data. The sample of children with useable data for each SRT test is depicted in Figure 1.

The SRT Battery was administered by trained research coordinators, as part of a home assessment visit when the children were between 48 and 50 months old. Upon arrival, research coordinators explained the procedure to caregivers and took consent. As English is the primary language used in education environments in Singapore, the home assessment visits were conducted entirely in English with the exception of non-test related communication (e.g., during warm-up) (Law et al., 2021).

The SRT Battery used in the current study was adapted from the Maternal Adversity, Vulnerability and Neurodevelopment (MAVAN) study based in Canada (O’Donnell et al., 2014). It should be noted that the measures of school readiness in this study will focus on cognitive school readiness skills which are more academic in nature, namely vocabulary, phonology, numeracy, general cognition, visual working memory and executive functioning. To summarize, the SRT Battery consists of measures in the language [Peabody Picture Vocabulary Test (PPVT), Comprehensive Test of Phonological Processing 2 (CTOPP-2)], Numeracy [Number Knowledge Test (NKT)] general cognition [Lollipop test], and memory [Visually Cued Recall Test (VCR), Random Object Span Test (ROST)] domains. With the exception of PPVT, which was always administered as the first test, the order of all other tests was counterbalanced across children.

Each test measure will be described briefly below. A detailed account can be found in the Supplementary Appendix. Further information of the SRT battery can also be found in previous publications (Lai et al., 2019; Law et al., 2021).

#### Language - Peabody Picture Vocabulary Test (PPVT)

The Peabody Picture Vocabulary Test (Fourth Edition, PPVT-4) is an achievement test of receptive vocabulary and provides an estimate of verbal ability (Dunn and Dunn, 2007). The PPVT-4 consists of 19 item sets with 12 target words in each item set. For each item set, the research coordinator began by showing the child a stimulus page with four pictures and saying a target word. The child then has to point to the picture that they feel corresponds with the target word.

#### Language - Comprehensive Test of Phonological Processing 2 (CTOPP-2)

The Comprehensive Test of Phonological Processing-2 (CTOPP-2) is an assessment tool used to test reading skills and phonological processing (Wagner et al., 1999) for ages 4–24 years old. Three out of the 10 CTOPP-2 subtests that focused on phonological awareness were administered, namely: Elision, Blending words and Sound matching.

The Elision subtest measures ability to identify parts (e.g., phonological segments) of a spoken word. The Blending Words subtest measures the ability to integrate sounds to found words. The Sound Matching subtest measures the ability to match words with the same initial or last sounds.

#### Numeracy - Number Knowledge Test (NKT)

The Number Knowledge Test is used to assess individual differences in number knowledge (Okamoto and Case, 1996). The test consisted of two levels (Level 0 and Level 1) with items of increasing difficulty.

In Level 0, the child was assessed based on his/her ability to count and quantify small sets. Three or more correct items (out of 5) at Level 0 was required for the child to move on to Level 1 and failure to do so resulted in the test being terminated at Level 0 (i.e., the child would have a score of zero for Level 1). Level 1 consisted of 13 questions on number sequence and simple arithmetic problems.

#### General cognition - Lollipop test

The Lollipop test is a well-validated diagnostic screening tool designed to assess different cognitive skill sets (Chew and Morris, 1984, 1987, 1989). The Lollipop test consists of four subtests: (1) Identification of Colors and Shapes, and Copying Shapes [Colors and Shapes], (2) Picture description, Position and Spatial Recognition [Picture description], (3) Identification of Numbers and Counting [Numbers] and (4) Identification of Letters and Writing [Letters].

#### Memory - Visually Cued Recall Test (VCR)

The Visually Cued Recall Test (VCR) incorporates both the pictorial memory span task and delayed response task (Zelazo et al., 2002). The VCR was designed to assess short-term (working) memory span and visuospatial abilities related to early academic achievement (Zelazo et al., 2002; Wang and Saudino, 2013).

At the beginning of the task, the child was presented with a toy dog called Molo. Next, Molo would then use his nose to indicate what he likes on a card with an array of pictures. After a 1 s delay where the card was covered, the pictures were revealed again and the child the child was asked to point out the target items that of Molo likes.

#### Memory - Random Object Span Test (ROST)

The Random Object Span Test (ROST) is a test of executive function in children, modified from the Self-ordered Pointing Task (SOPT; Petrides and Milner, 1982). Each trial required children to select all the images in an array without re-selecting the same image twice. The position of the image array would change between trials, so that the child has to keep mental account of previous images selected within previous trials.

### Other data including covariate measures

Participant demographic information including ethnicity and maternal education were collected during enrollment. Birth weight (amongst other birth outcome data) were collected during delivery. Interviewer administered questionnaires about feeding practices were administered to mothers when the infants were at 3, 6, 9, 12, 15, 18, 24, 36, and 48 months. Detailed information about breastfeeding practices in the GUSTO sample have been reported previously (Cai et al., 2015). The above variables were selected as they are common covariates that are known to influence sleep and/or outcomes related to cognitive and language domains, especially in the GUSTO sample (Cai et al., 2015; Zhou et al., 2015; Tham et al., 2019; Jafar et al., 2021). In addition, recent work on the GUSTO cohort exploring the effects of sleep duration trajectories in early childhood cognitive development have also adjusted for the above set of covariates (Cai et al., 2022).

### Statistical analyses

Statistical analyses were carried out using SPSS Statistics version 23 (IBM). Participant characteristics were analyzed using Independent-samples *t*-tests (continuous measures) and chi-square tests (categorical measures). First, unadjusted Analysis of Variance (ANOVA) models were performed to assess associations between sleep trajectories and individual SRT tests – PPVT, CTOPP-2, NKT, Lollipop test, VCR and ROST (Unadjusted Model). Next, Analysis of Covariance (ANCOVA) models were performed to assess associations between sleep trajectories and above SRT tests (PPVT, CTOPP-2, NKT, Lollipop test, VCR and ROST) adjusting for effects of ethnicity, birth weight, maternal education and breastfeeding (Adjusted Model). Planned *post hoc* comparisons were conducted using Dunnett’s Multiple Comparison with the short trajectory group as the reference.

## Results

### Participant characteristics

Amongst the children who met the study inclusion criteria and took part in the SRT battery, participant characteristics of children with sleep trajectories (i.e., those included in the study analyses) were comparable to those without sleep trajectories in terms of ethnicity, sex, gestational age, birth weight, maternal age and maternal (highest) education (Table 1). However, children with sleep trajectories were breastfed longer.

**TABLE 1:** Summary of participant characteristics amongst children who took part in the school readiness test battery.

	Study sample – children with sleep trajectory data (*n* = 288)	Excluded – children without sleep trajectory data (*n* = 407)	*P*-value
Ethnicity, *n* (%)			0.14^#^
Chinese	173 (60.07%)	220 (54.05%)	
Malay	77 (26.74%)	101 (24.82%)	
Indian	38 (13.19%)	86 (21.13%)	
**Sex of child**			
Female, *n* (%)	141 (48.96%)	204 (50.12%)	0.81
Gestational age (weeks)	39.10 (1.03)	39.03 (1.00)	0.38
Birth weight (g)	3142 (400)	3161 (396)	0.51
Maternal age (years)	31.05 (5.96)	30.43 (5.08)	0.11
Maternal highest education, *n* (%)			0.11
Primary	11 (3.82%)	27 (6.63%)	
Secondary	63 (21.88%)	100 (24.57%)	
Diploma/Technical education	102 (35.42%)	134 (32.92%)	
University	109 (37.85%)	142 (34.89%)	
Missing data	3 (1.04%)	4 (0.98%)	
Duration of breastfeeding, *n* (%)			0.03
<1 month	54 (18.75%)	100 (24.57%)	
1 to <3 months	52 (18.06%)	66 (16.22%)	
3 to <6 months	46 (15.97%)	69 (16.95%)	
6 to <12 months	58 (20.14%)	70 (17.20%)	
≥12 months	74 (25.69%)	80 (19.66%)	
Missing data	4 (1.38%)	22 (5.41%)	

Data presented as *n* (%) or mean (SD).

^#^Comparing Chinese with non-Chinese ethnicity.

### Total sleep trajectories and academic SRT

The overall associations between total sleep trajectories and individual SRT tests are presented in Table 2.

**TABLE 2:** Association between total sleep trajectories and academic SRT performance.

	Unadjusted^1^ model				Adjusted^2^ model		
	**Short variable** **(reference group)**	**Moderate consistent**	**Long variable**	**Long consistent**	**Moderate consistent**	**Long variable**	**Long consistent**
**Language**							
Receptive vocabulary (PPVT)^3^	84.41 ± 18.79	89.99 ± 17.93	85.70 ± 17.49	91.64 ± 16.59*	5.56 (−0.81–11.93)	0.84 (−6.36–8.04)	7.11 (0.63–13.60)
Phonological awareness (CTOPP-2)^4^	92.49 ± 7.28	95.80 ± 8.27	93.94 ± 8.35	96.04 ± 9.13*	3.53 (0.30–6.76)	1.06 (−2.66–4.77)	3.49 (0.23–6.74)
**Numeracy**							
Number Knowledge Test	5.00 ± 3.33	6.33 ± 3.74	5.48 ± 3.05	6.37 ± 3.88	1.37 (0.03–2.71)	0.38 (−1.13–1.90)	1.40 (0.06–2.74)
**General cognition**							
Lollipop test	40.29 ± 14.63	44.16 ± 12.45	43.02 ± 12.92	43.09 ± 13.55	4.29 (−0.61–9.19)	2.33 (−3.16–7.82)	2.88 (−2.04–7.79)
**Memory**							
Visually Cued Recall Test	3.16 ± 1.74	3.43 ± 1.41	3.30 ± 1.38	3.13 ± 1.58	0.26 (−0.33–0.85)	0.14 (−0.53–0.80)	−0.04 (−0.64–0.55)
Random Object Span Test	4.89 ± 3.26	5.30 ± 2.96	4.17 ± 2.77	5.32 ± 2.77	0.38 (−0.78–1.54)	−0.79 (−2.09–0.52)	0.39 (−0.78–1.55)

^1^Values are unadjusted means ± SDs.

^2^Adjusted mean difference in SRT scores (95% confidence interval) from reference group (short variable), adjusted for maternal education, birth weight, breastfeeding duration and ethnicity.

^3^Peabody Picture Vocabulary Test.

^4^Comprehensive Test of Phonological Processing 2.

*p < 0.05 for mean difference against reference group (short variable).

Bold values depict significant findings.

For the unadjusted model, there was a significant main effect of total sleep trajectory groups for PPVT scores, *F*(3,281) = 2.67, *p* = 0.048. Planned comparisons between trajectory groups revealed that children within the short variable group had significantly lower PPVT scores than children in the long consistent group (*p* = 0.04). There were no significant differences in PPVT scores between the short variable group and the long variable group (*p* = 0.96) or moderate consistent group (*p* = 0.13).

However, there was no significant effect of sleep trajectory groups on PPVT scores in the model which adjusted for maternal education, birth weight, breastfeeding duration and ethnicity (adjusted model), *F*(3,260) = 1.27, *p* = 0.29.

There was also a significant effect of total sleep trajectory group on the CTOPP-2 phonological awareness scores in the unadjusted model, *F*(3,263) = 2.73, *p* = 0.044, whereby the short variable group had significantly lower phonological awareness scores than both the long consistent (*p* = 0.04) group. In contrast, there were no group differences between the short variable group and both the moderate consistent (*p* = 0.05) and the long variable (*p* = 0.69) groups.

Similar to the PPVT analyses, there was no significant effect of sleep trajectory groups in the adjusted model, *F*(3,242) = 1.60, *p* = 0.19.

There were no significant effects of sleep trajectory group for the NKT, Lollipop test, VCR and ROST in both the unadjusted and adjusted models (all *p*’s > 0.05).

### Night sleep trajectories and academic SRT

The overall associations between night sleep trajectories and individual SRT tests are presented in Table 3.

**TABLE 3:** Association between night sleep trajectories and SRT performance.

	Unadjusted^1^ model			Adjusted^2^ model	
	**Short variable** **(reference group)**	**Moderate consistent**	**Long consistent**	**Moderate consistent**	**Long consistent**
**Language**					
Receptive vocabulary (PPVT)^3^	85.41 ± 19.93	89.69 ± 18.30	89.04 ± 15.26	4.80 (−0.50–10.10)	3.89 (−1.72–9.50)
Phonological awareness (CTOPP-2)^4^	94.01 ± 8.03	94.80 ± 8.10	95.59 ± 9.13	1.12 (−1.54–3.77)	1.82 (−1.01–4.65)
**Numeracy**					
Number Knowledge Test	5.12 ± 3.56	5.74 ± 3.43	6.65 ± 3.71**	0.55 (−0.54–1.64)	**1.53 (0.38**–2.69)**
**General cognition**					
Lollipop test	40.12 ± 14.48	44.03 ± 12.73	43.52 ± 12.98	3.93 (−0.08–7.93)	3.63 (−0.60–7.85)
**Memory**					
Visually Cued Recall Test	3.18 ± 1.63	3.27 ± 1.52	3.34 ± 1.45	0.12 (−0.36–0.60)	0.19 (−0.32–0.70)
Random Object Span Test	4.94 ± 3.27	5.08 ± 3.06	4.84 ± 2.54	0.13 (−0.82–1.09)	−0.22 (−1.23–0.79)

^1^Values are unadjusted means ± SDs.

^2^Adjusted mean difference in SRT scores (95% confidence interval) from reference group (short variable), adjusted for maternal education, birth weight, breastfeeding duration and ethnicity.

^3^Peabody Picture Vocabulary Test.

^4^Comprehensive Test of Phonological Processing 2.

**p < 0.01 for mean difference against reference group (short variable).

Bold values depict significant findings.

Analyses revealed a significant effect of night sleep trajectory groups for the Number Knowledge Test (NKT) for the unadjusted model, *F*(2,288) = 4.11, *p* = 0.02. Children with short variable night sleep trajectories had significantly lower NKT scores compared to children with long consistent trajectories (*p* = 0.009) but not when compared to children with moderate consistent sleep trajectories (*p* = 0.37).

In addition, the effect of night sleep trajectory on NKT performance remained significant in the adjusted model, *F*(2,267) = 3.79, *p* = 0.02. After controlling for the effects of maternal education, birth weight, breastfeeding duration and ethnicity, children with short variable night sleep continue to display lower NKT scores compared to their counterparts with long consistent sleep (*p* = 0.007). Similar to findings in the unadjusted model, there were no significant differences in NKT performance between children with short variable and moderate consistent night sleep (*p* = 0.42).

There were no significant effects of night sleep trajectory group for the other school readiness language or cognitive based tests in both the unadjusted and adjusted models (all *p*’s > 0.05).

### Day sleep trajectories and academic SRT

In contrast to total sleep and night sleep trajectory findings, there were no significant relations between day sleep trajectories and cognitive school readiness (Table 4) across all domains in both the unadjusted and adjusted models (all *p*’s > 0.05).

**TABLE 4:** Association between day sleep trajectories and SRT performance.

	Unadjusted^1^ model				Adjusted^2^ model		
	**Short consistent** **(reference group)**	**Moderate consistent**	**Long variable**	**Long consistent**	**Moderate consistent**	**Long VARIABLE**	**Long consistent**
**Language**							
Receptive vocabulary (PPVT)^3^	87.71 ± 18.90	88.57 ± 18.59	84.78 ± 14.68	91.25 ± 17.91	0.67 (−5.57–6.91)	−3.51 (−10.66–3.64)	3.32 (−3.61–10.25)
Phonological awareness (CTOPP-2)^4^	94.15 ± 8.67	95.63 ± 8.83	93.32 ± 8.30	95.33 ± 7.29	1.39 (−1.76–4.54)	−1.36 (−4.94–2.22)	0.92 (−2.59–4.44)
**Numeracy**							
Number Knowledge Test	5.68 ± 4.17	6.34 ± 3.60	5.34 ± 3.08	5.68 ± 3.32	0.61 (−0.70–1.92)	−0.48 (−1.97–1.00)	0.06 (−1.40–1.51)
**General cognition**							
Lollipop test	41.58 ± 14.32	42.64 ± 12.76	42.32 ± 14.23	44.28 ± 12.75	0.87 (−3.93–5.67)	0.05 (−5.39–5.48)	2.48 (−2.86–7.81)
**Memory**							
Visually Cued Recall Test	3.50 ± 1.70	3.28 ± 1.46	3.22 ± 1.54	3.02 ± 1.43	−0.25 (−0.82–0.33)	−0.30 (−0.95–0.34)	−0.52 (−1.16–0.12)
Random Object Span Test	5.49 ± 2.85	4.86 ± 2.97	4.50 ± 3.05	5.13 ± 2.95	−0.74 (−1.87–0.40)	−1.08 (−2.37–0.20)	−0.48 (−1.73–0.78)

^1^Values are unadjusted means ± SDs.

^2^Adjusted mean difference in SRT scores (95% confidence interval) from reference group (short consistent), adjusted for maternal education, birth weight, breastfeeding duration and ethnicity.

^3^Peabody Picture Vocabulary Test.

^4^Comprehensive Test of Phonological Processing 2.

## Discussion

To the best of our knowledge, this is the first study to explore how differences in child sleep trajectories across the first 4.5 years of life are associated with subsequent measures of academic school readiness at age 4. Overall, our findings suggest that early childhood sleep pattern is a modest variable associated with SRT. Short variable sleep trajectories was related to poorer numeracy performance at 4 years of age compared to having longer (duration) and more consistent (less variable) sleep trajectories when adjusted for confounders.

Our key finding was that children with short variable night sleep trajectories had poorer performance on the Number Knowledge Test (NKT) compared to their peers with longer and consistent night sleep trajectories. This is consistent with existing studies in school-aged children, whereby amongst 7–9 year old school-aged children, those with long sleep duration had significantly better numeracy performance than their peers who were classified as having typical sleep, variable bedtimes or short sleep (Blunden et al., 2018). Moreover, by experimentally altering 8–12 year old school-aged children’s habitual sleep duration into short sleep (bedtimes an hour earlier) or long sleep (bedtimes an hour later), researchers found that children had better Math fluency performance when they were in the long sleep condition compared to the short sleep condition. In addition to vocabulary, sleep is also crucial for the consolidation other forms of declarative/explicit memory that do not directly involve language (Wilhelm et al., 2008; Seehagen et al., 2014). Our current findings support this as the children with short night sleep trajectories would have fewer opportunities to consolidate newly learnt numerical concepts compared to their peers with longer sleep duration trajectories.

For the unadjusted model, children with short variable total sleep trajectories had lower receptive vocabulary scores (as measured with the PPVT) and lower CTOPP-2 phonological awareness scores compared to children with long consistent trajectories. Our findings support existing studies in children (2.5–6 years) that highlight that children with short night sleep trajectories were more than 3 times at risk for low PPVT-R receptive vocabulary performance compared to children with longer 11-h sleep trajectories (Touchette et al., 2007). Although there is limited work on sleep duration and phonological awareness, previous research has shown that school-aged children with sleep problems such as sleep disordered breathing perform poorer in measures of phonological awareness compared to age-matched controls (Brien et al., 2004a,b).

Unlike the SRT tasks that assessed aspects of language and numeracy development, there were no significant associations between sleep trajectories and general cognition or working memory or executive functions, namely accessed *via* the Lollipop test, the Visually Cued Recall Test (VCR) and the Random Object Span Test (ROST). Even though there have many studies on highlighting an association between sleep and general cognition or working memory/executive functioning in school-aged children, the associations are specific to certain aspects of cognition or working memory/executive function or sleep (Short et al., 2018). For example, VCR was designed to assess short-term visuospatial working memory span and previous studies in kindergarten found that although short sleep duration was related to poorer verbal working memory performance, there was no relation between sleep duration and visuospatial working memory performance (Cho et al., 2015). In a meta-analysis of studies exploring sleep duration and cognitive processes in children aged 5–13 years, researchers did not find a significant association between sleep duration and executive function (Short et al., 2018). Similarly, in teenagers, executive function was related to self-reported levels of sleepiness but not sleep duration (Anderson et al., 2009). Finally, it should be noted that we did not observe any associations with day sleep trajectories. However, previous studies also do not observe any association between day sleep trajectories and developmental outcomes in children this age (Smithson et al., 2018).

Notably, this study is the first study to examine variability in addition to duration of sleep in relation to academic/cognitive school readiness, allowing for a more developmental pattern approach. Children with consistent sleep trajectories display smaller between individual variability (i.e., their sleep patterns are more similar to typical sleep patterns in the analyzed/sample population) than children with variable sleep trajectories (Tham et al., 2021). An interesting finding in the current study is that children with long variable total sleep trajectories did not display significantly better SRT performance compared to children with short variable trajectories. This highlights that both variability and duration of sleep trajectories are important toward SRT performance, i.e., children who have both longer and more consistent sleep trajectories have better performance in numeracy, whereas children who only display longer sleep trajectories do not have equivalent “advantages.”

However, some study limitations should be noted. In particular, less than half of the children with SRT data also had sleep trajectory data: out of the 695 children who met the study inclusion criteria and had at least one useable SRT task data, only 288 children (41%) had sleep trajectory data and were included in the study analyses. Still, with the exception of the duration of breastfeeding, all other participant characteristics of the study sample where comparable to their peers who were excluded in the analyses. Also, we adjusted for these characteristics, inclusive breastfeeding, in our analyses. Next, it should be noted that the relation between total sleep and language was only present in the unadjusted models and not in the adjusted models. This may be because such tasks as typically driven by small effects (effect sizes), and such effects are no longer present in our adjusted analyses. Further research with a larger sample of children would be helpful to access the above hypothesis. Sleep duration at 3–24 months was accessed using the BISQ and at 54 months using the CSHQ. This change in questionnaires was to ensure that measures were age-appropriate for the relevant time point (Owens et al., 2000; Sadeh, 2004). However, it is possible that methodological differences between the questionnaires could have affected caregiver responses. In addition the sleep trajectories were derived from caregiver-reported sleep durations, which may be subjective in nature. Even though both the BISQ and CSHQ have either been validated using more objective measures like actigraphy or assessed to be “well-established” questionnaires by the American Psychological Association (APA) Division 54 Evidence-Based Assessment (EBA) Task Force (Sadeh, 2004; Lewandowski et al., 2011), it would also be useful to replicate this current analyses with more objective sleep measures like actigraphy or polysomnography. The SRT battery used in this study focused on academic school readiness, namely the domains of language, numeracy, general cognition and memory. Apart from the above domains, non-academic-related factors such as socio-emotional and physical development, and temperament are also important aspect of school readiness (High, 2008). Therefore, it would be useful for future studies to also explore the relations between longitudinal sleep duration trajectories and other domains of school readiness, for example physical development and temperament. Although we have adjusted for covariate variables that have been previously shown to affect sleep and/or cognition in our GUSTO sample, it is possible that other variables such as maternal age may also be confounding variables in the analyses.

Despite the above limitations, the study also has important strengths. For example, we included a broad range of school readiness tests that covered domains of language, numeracy and cognitive functions. Existing school readiness studies have focused on point-estimate sleep duration data during childhood, whereas we examined longitudinal sleep across multiple time points in early infancy until early childhood. Another novel aspect of the study is that it explored between-individual variability of sleep in addition to differences in sleep duration.

## Conclusion

In conclusion, this is the first study to explore the associations between longitudinal sleep duration and between-individual variation in sleep trajectories and academic school readiness performance in preschool children. Children with short variable sleep trajectories displayed poorer performance in school readiness tests associated with numeracy (adjusted models) and language (unadjusted models) compared to their peers with longer and more consistent sleep trajectories. In contrast, there were no associations between sleep trajectories and SRT tasks that assessed general cognitive or memory functions. Overall, findings suggest that individual differences in longitudinal sleep duration patterns from as early as 3 months of age may be associated with aspects of academic school readiness at 4 years of age. This is important, as early school readiness, particularly the domains of language and mathematics, is a key predictor of subsequent academic achievement. Further studies with larger sample sizes are needed to address the issue of effect sizes.

## Data availability statement

The raw data supporting the conclusions of this article will be made available by the authors, without undue reservation.

## Ethics statement

The studies involving human participants were reviewed and approved by the National Health Care Group Domain Specific Review Board and the SingHealth Centralized Institutional Review Board. Written informed consent to participate in this study was provided by the participants’ legal guardian/next of kin.

## Author contributions

ET did the literature review, conceptualized the idea for the manuscript, analyzed the data, wrote the manuscript, and involved in the data cleaning of the sleep data used. H-YX and XF involved in generating the sleep trajectories and reviewed the manuscript. RG involved in supervising the IHPC team and reviewed the manuscript. LP-CS involved in supervising data collection and reviewed the manuscript. PG, Y-SC, FY, and MM conceptualized the cohort study, involved in supervising the data collection, and reviewed the manuscript. OT, JG, and DY-TG involved in the design of the questionnaire or protocol used in the tasks as well as in the data collection and reviewed the manuscript. NS helped with the manuscript preparation and conceptualized the idea for the manuscript. SC involved in the data cleaning of the sleep data used, conceptualized the idea for the manuscript, and reviewed the manuscript. BB involved in supervising the data collection, conceptualized the idea for the manuscript, and reviewed the manuscript. All authors contributed to the article and approved the submitted version.

## Footnotes

1
https://www.singstat.gov.sg/

## References

[B1] Anderson B. Storfer-Isser A. Taylor H. G. Rosen C. L. Redline S. (2009). Associations of executive function with sleepiness and sleep duration in adolescents. *Pediatrics* 123 e701–e707. 10.1542/peds.2008-1182 19336360 PMC4430079

[B2] Blunden S. Magee C. Attard K. Clarkson L. Caputi P. Skinner T. (2018). Sleep schedules and school performance in Indigenous Australian children. *Sleep Health* 4 135–140. 10.1016/j.sleh.2017.12.006 29555125

[B3] Brien L. M. O. Mervis C. B. Holbrook C. R. Bruner J. L. Smith N. H. Nechia M. (2004a). Neurobehavioral correlates of sleep-disordered breathing in children. *J. Sleep Res.* 13 165–172. 15175097 10.1111/j.1365-2869.2004.00395.x

[B4] Brien L. M. O. Raffield T. Gozal D. (2004b). Neurobehavioral implications of habitual snoring in children. *Pediatrics* 114 44–49. 10.1542/peds.114.1.44 15231906

[B5] Bruni O. Baumgartner E. Sette S. Ancona M. Caso G. Di Cosimo M. E. (2014). Longitudinal study of sleep behavior in normal infants during the first year of life. *J. Clin. Sleep Med.* 10 1119–1127. 10.5664/jcsm.4114 25317093 PMC4173090

[B6] Cai S. Tham E. K. H. Xu H. Y. Fu X. Goh R. S. M. Gluckman P. D. (2022). Trajectories of reported sleep duration associate with early childhood cognitive development. *Sleep* 46:zsac264. 10.1093/sleep/zsac264 36355436 PMC9905782

[B7] Cai S. Pang W. W. Low Y. L. Sim L. W. Sam S. C. Bruntraeger M. B. (2015). Infant feeding effects on early neurocognitive development in Asian children. *Am. J. Clin. Nutr.* 101 326–336. 10.3945/ajcn.114.095414 25646330

[B8] Chew A. L. Morris J. D. (1984). Validation of the lollipop test: A diagnostic screening test of school readiness. *Educ. Psychol. Meas.* 44 987–991. 10.1177/0013164484444022

[B9] Chew A. L. Morris J. D. (1987). Investigation of the lollipop test as a pre-kindergarten screening instrument. *Educ. Psychol. Meas.* 47 467–471. 10.1177/0013164487472019

[B10] Chew A. L. Morris J. D. (1989). Predicting later academic achievement from kindergarten scores on the metropolitan readiness tests and the lollipop test. *Educ. Psychol. Meas.* 49 461–465. 10.1177/0013164489492019

[B11] Cho M. Quach J. Anderson P. Mensah F. Wake M. Roberts G. (2015). Poor sleep and lower working memory in grade 1 children: Cross-sectional, population-based study. *Acad. Pediatr.* 15 111–116. 10.1016/j.acap.2014.06.021 25528129

[B12] Currie J. (2005). Health disparities and gaps in school readiness. *Future Child.* 15 117–138. 10.1353/foc.2005.0002 16130544

[B13] Duncan G. J. Dowsett C. J. Claessens A. Magnuson K. Huston A. C. Klebanov P. (2007). School readiness and later achievement. *Dev. Psychol.* 43 1428–1446. 10.1037/0012-1649.43.6.1428 18020822

[B14] Duncan G. J. Engel M. Heckman J. Hill H. Kalil A. Lipsey M. (2006). *Kindergarten skills and fifth grade achievement: Evidence from the ECLS-K Amy claessens.* Washington, DC: National Association for the Education of Young Children.

[B15] Dunn L. M. Dunn D. M. (2007). *PPVT-4 manual.* Bloomington, MN: NCS Pearson, Inc.

[B16] Galland B. C. Taylor B. J. Elder D. E. Herbison P. (2012). Normal sleep patterns in infants and children: A systematic review of observational studies. *Sleep Med. Rev.* 16 213–222. 10.1016/j.smrv.2011.06.001 21784676

[B17] High P. C. (2008). School readiness. *Pediatrics* 121 e1008–e1015. 10.1542/peds.2008-0079 18381499

[B18] Iglowstein I. Jenni O. G. Molinari L. Largo R. H. (2003). Sleep duration from infancy to adolescence: Reference values and generational trends. *Pediatrics* 111 302–307. 10.1542/peds.111.2.302 12563055

[B19] Jafar N. K. Tham E. K. Pang W. W. Fok D. Chua M. C. Teoh O. H. (2021). Association between breastfeeding and sleep patterns in infants and preschool children. *Am. J. Clin. Nutr.* 114 1986–1996. 10.1093/ajcn/nqab297 34582549

[B20] Lai J. S. Cai S. Feng L. Shek L. P. Yap F. Tan K. H. (2019). Associations of maternal zinc and magnesium with offspring learning abilities and cognitive development at 4 years in GUSTO. *Nutr. Neurosci.* 24 467–476. 10.1080/1028415x.2019.1643624 31331255

[B21] Law E. C. Aishworiya R. Cai S. Bouvette-Turcot A.-A. Broekman B. F. P. Chen H. (2021). Income disparity in school readiness and the mediating role of perinatal maternal mental health: A longitudinal birth cohort study. *Epidemiol. Psychiatr. Sci.* 30:e6. 10.1017/S204579602000102X 33416045 PMC8057379

[B22] Lewandowski A. S. Toliver-Sokol M. Palermo T. M. (2011). Evidence-based review of subjective pediatric sleep measures. *J. Pediatr. Psychol.* 36 780–793. 10.1093/jpepsy/jsq119 21227912 PMC3146754

[B23] Mistry R. S. Benner A. D. Biesanz J. C. Clark S. L. Howes C. (2010). Family and social risk, and parental investments during the early childhood years as predictors of low-income children’s school readiness outcomes. *Early Child. Res. Q.* 25 432–449. 10.1016/j.ecresq.2010.01.002

[B24] O’Donnell K. A. Gaudreau H. Colalillo S. Steiner M. Atkinson L. Moss E. (2014). The maternal adversity, vulnerability and neurodevelopment project: Theory and methodology. *Can. J. Psychiatry* 59 497–508. 10.1177/070674371405900906 25565695 PMC4168812

[B25] Okamoto Y. Case R. (1996). Exploring the microstructure of children’s central conceptual structures in the domain of number. *Monogr. Soc. Res. Child Dev.* 61 27–58. 10.1111/j.1540-5834.1996.tb00536.x 8657168

[B26] Owens J. A. Spirito A. McGuinn M. (2000). The children’s sleep habits questionnaire (CSHQ): Psychometric properties of a survey instrument for school-aged children. *Sleep* 23 1043–1051. 10.1093/sleep/23.8.1d 11145319

[B27] Pekdogan S. Akgul E. (2016). Preschool children’s school readiness. *Int. Educ. Stud.* 10:144. 10.5539/ies.v10n1p144

[B28] Petrides M. Milner B. (1982). Deficits on subject-ordered tasks after frontal and temporal-lobe lesions in man. *Neuropsychologia* 20 249–262. 10.1016/0028-3932(82)90100-2 7121793

[B29] Plancoulaine S. Reynaud E. Forhan A. Lioret S. Heude B. Charles M. A. (2018). Night sleep duration trajectories and associated factors among preschool children from the EDEN cohort. *Sleep Med.* 48 194–201. 30008301 10.1016/j.sleep.2018.03.030

[B30] Ravid S. Afek I. Suraiya S. Shahar E. Pillar G. (2009). Kindergarten children’s failure to qualify for first grade could result from sleep disturbances. *J. Child Neurol.* 24 816–822. 10.1177/0883073808330766 19189933

[B31] Sadeh A. (2004). A brief screening questionnaire for infant sleep problems: Validation and findings for an internet sample. *Pediatrics* 113 e570–e577. 10.1542/peds.113.6.e570 15173539

[B32] Sadeh A. Mindell J. A. Luedtke K. Wiegand B. (2009). Sleep and sleep ecology in the first 3 years: A web-based study. *J. Sleep Res.* 18 60–73. 10.1111/j.1365-2869.2008.00699.x 19021850

[B33] Seehagen S. Konrad C. Herbert J. S. Schneider S. (2014). Timely sleep facilitates declarative memory consolidation in infants. *Proc. Natl. Acad. Sci. U.S.A.* 112 1625–1629. 10.1073/pnas.1414000112 25583469 PMC4321279

[B34] Short M. A. Blunden S. Rigney G. Matricciani L. Coussens S. M. Reynolds C. (2018). Cognition and objectively measured sleep duration in children: A systematic review and meta-analysis. *Sleep Health* 4 292–300. 10.1016/j.sleh.2018.02.004 29776624

[B35] Smithson L. Baird T. Tamana S. K. Lau A. Mariasine J. Chikuma J. (2018). Shorter sleep duration is associated with reduced cognitive development at two years of age. *Sleep Med.* 48 131–139. 10.1016/j.sleep.2018.04.005 29906629

[B36] Soh S.-E. Tint M. T. Gluckman P. D. Godfrey K. M. Rifkin-Graboi A. Chan Y. H. (2013). Cohort profile: Growing up in Singapore towards healthy outcomes (GUSTO) birth cohort study. *Int. J. Epidemiol.* 43 1401–1409. 10.1093/ije/dyt125 23912809

[B37] Tham E. K. H. Richmond J. Gooley J. J. Jafar N. K. Chong Y.-S. Yap F. (2019). Variations in habitual sleep and relational memory in 6-month-olds. *Sleep Health* 5 257–265. 10.1016/j.sleh.2018.12.007 31208709

[B38] Tham E. K. H. Xu H.-Y. Fu X. Schneider N. Goh D. Y. T. Lek N. (2021). Variations in longitudinal sleep duration trajectories from infancy to early childhood. *Sleep Health* 7 56–64. 10.1016/j.sleh.2020.06.007 32843312

[B39] Touchette E. Côté S. M. Petit D. Liu X. Boivin M. Falissard B. (2009). Short nighttime sleep-duration and hyperactivity trajectories in early childhood. *Pediatrics* 124 e985–e993. 10.1542/peds.2008-2005 19841107

[B40] Touchette E. Dionne G. Forget-Dubois N. Petit D. Pérusse D. Falissard B. (2013). Genetic and environmental influences on daytime and nighttime sleep duration in early childhood. *Pediatrics* 131 e1874–e1880. 10.1542/peds.2012-2284 23713101

[B41] Touchette É. Petit D. Séguin J. Boivin M. (2007). Associations between sleep duration patterns and behavioral/cognitive functioning at school entry. *Sleep* 30 1213–1219. 10.1093/sleep/30.9.1213 17910393 PMC1978413

[B42] Tso W. Rao N. Jiang F. Li A. M. Lee S. Ho F. K. (2016). Sleep duration and school readiness of Chinese preschool children. *J. Pediatr.* 169 266–271. 10.1016/j.jpeds.2015.10.064 26608085

[B43] Wagner R. Torgesen J. Rashotte C. Pearson N. A. (1999). *Comprehensive test of phonological processing*, 2nd Edn. Austin, TX: Pro-ed.

[B44] Wang M. Saudino K. J. (2013). Genetic and environmental influences on individual differences in emotion regulation and its relation to working memory in toddlerhood. *Emotion* 13 1055–1067. 10.1037/a0033784 24098922 PMC4108294

[B45] Wilhelm I. Diekelmann S. Born J. (2008). Sleep in children improves memory performance on declarative but not procedural tasks. *Learn. Mem.* 15 373–377. 10.1101/lm.803708 18441295

[B46] Williams P. G. Lerner M. A. Sells J. Alderman S. L. Hashikawa A. Mendelsohn A. (2019). School readiness. *Pediatrics* 144 91–96. 10.1542/peds.2019-1766 31331984

[B47] Zelazo P. D. Jacques S. Burack J. A. Frye D. (2002). The relation between theory of mind and rule use: Evidence from persons with autism-spectrum disorders. *Infant Child Dev.* 11 171–195. 10.1002/icd.304

[B48] Zhou Y. Aris I. M. Tan S. S. Cai S. Tint M. T. Krishnaswamy G. (2015). Sleep duration and growth outcomes across the first two years of life in the GUSTO study. *Sleep Med.* 16 1281–1286. 10.1016/j.sleep.2015.07.006 26429758

