# Supplementary material for: Associations between sleep trajectories up to 54 months and cognitive school readiness in 4 year old preschool children
Source: Front Psychol. 2023 Mar 28;14:1136448. doi: 10.3389/fpsyg.2023.1136448 (PMC10086425; doi:10.3389/fpsyg.2023.1136448)
Supplement: Supplementary file 1 [file Data_Sheet_1.docx]

**Supplementary Materials**

**School Readiness Test (SRT) Battery**

**Peabody Picture Vocabulary Test (PPVT)**

The PPVT consists of two forms (Form A and Form B; for multiple administrations). Form A was used for this study. At the start of the test, Training Page B (Ages 4 onwards) was shown to the child as the practice set. If the child managed to answer two out of four questions correctly, the experimenter began the task with Set 2 (for Age 4). If, however, the child did not manage to achieve 2 out of 4 questions correctly, the experimenter returned to Training Page A (Ages 2:6 Through 3:11) for another round of practice before beginning the task at Set 1 (for Age 2:6 – 3:11). A Basal Set was established when the child made one or zero errors in the first set. If two or more errors were made, the research coordinator returned to a previous set in order to establish a Basal Set.

The task was terminated when eight or more errors were made in an item set (i.e. Ceiling Set). A raw score was calculated by subtracting the total number of errors across all sets from the last item number in the Ceiling Set. A PPVT standard score was then derived from the raw score and child’s age.

**Comprehensive Test of Phonological Pocessing-2 (CTOPP-2)**

**Elison**. The researcher coordinator would instruct the child: “Say ‘toothbrush’’. Now say ‘toothbrush’ without saying ‘tooth’”. The task gets progressively harder where even smaller parts of a word (consonant) is taken away, for example saying cat without the /k/. The research coordinator was allowed to repeat each test item twice. There were 34 items in this subtest where corrective feedback was given until item 14, after which no feedback was allowed.

**Blending Words.** For each item, syllable sounds were presented to the child via a CD recording. The child was then required to put together the syllable sounds to form a word. Out of the 33 items for this subset, corrective feedback was allowed until item 12.

**Sound Matching.** A picture book was used to present the 26 item-sets for this subtest. For each item set, the research coordinator would say a target word and list would the potential answers from the stimuli set. The child would then select the ‘matching’ words (with the same sounds) from the picture book either by pointing to the correct pictures or saying the words aloud. The Sound matching subtest has two parts. The first part requires the child to match a word to first sound, for example, “which of these picture words starts with the /s/ sound like sock? Sun or bear?”. The second part requires child to match a word to last sound: which of these words ends with the /n/ sound like can? Pot or sun? When listing the stimuli in each item-set, once the target word is pronounced, the research coordinator must make a clear pause for 1 second before pronouncing the alternative answers. Each item could only be repeated once.

For each of the subtest, a correct response was scored as ‘1’ and an incorrect response was scored as ‘0’. Each subtest was terminated when the child made three consecutive incorrect responses. The raw scores from each subtest were computed into scaled scores based on the child’s date of birth. The scaled scores for each subtest were then summed up to compute an individual phonological awareness composite score.

**Lollipop Test**

For each item, a stimulus card was presented to the child and responses were recorded on a response sheet, with the exception of the following questions: under subtest 1 [Colours and Shapes], where the child was required to draw shapes on a piece of paper, and in subtest 4 [Letters], where the child was asked to illustrate alphabets and his/her name. A prompt was given if the child did not respond or provide an answer to the question.

Each item was scored either a pass (1 point) or fail (0 point), with some exceptions e.g. name writing, where up to 5 points could be awarded (Chew & Morris, 1987). Additionally, there is no termination rule in this test. Four scores were derived from the four subtests. The total Lollipop score was then computed by adding the scores of the four subtests. Higher Lollipop subtest and total scores indicated better performance.

**Number Knowledge Test (NKT)**

The NKT began with a practice question where the child was required to count from one to 10 aloud. This orientated the child to the nature of the test and allowed the child to warm up. The research coordinator then proceeded to the Level 0 of the test, which consists of five items. For Level 1, the child was required to deal with quantities that cannot be seen or touched and requires the child to imagine. In other words, solving items in this level required children to rely on a "mental counting line" inside their heads.

Each item was scored based on a pass (1 point) or fail (0 point) criteria. The test was terminated when the child obtained three consecutive mistakes. A total NKT score was derived from the addition of all successful test items.

**Visually Cued Recall Task (VCR)**

In each trial, the child had to constantly update their mental representations of Molo’s favourite items and retain visual images and spatial locations of the items across the short delay (Wang & Saudino, 2013). Feedback was given during practice trials, whereas no feedback was given during the test trials. During the process in the identification of the items, it is essential for the child to recognize and focus their attention on the specified items, and to differentiate the target items from other appealing distractors on the card (Wang & Saudino, 2013). For each trial, the child was prompted once if he/she missed out an item.

VCR consists of 2 practice trials and 11 test trials (9 levels with 12 items and 2 levels with 18 items), where the child had to remember increasing number of target items on each level. The test was terminated when two or more errors were made on two consecutive trials. The total score was derived from the addition of all successful test trials.

**Random Object Span Task (ROST)**

**Practice Trials.** When the child was presented with the first stimulus card for that set, the experimenter would tell the child to “Choose a picture.” After flipping to the subsequent stimulus card, the experimenter would say “Another.” Since the aim of the practice session was to ensure that the child understood the instructions clearly (i.e. to select all the images in an array without re-selecting the same image twice), feedback was given to the child. Once the child completed two consecutive practice trials correctly, they were allowed to move on to the first test trial. If the child failed on all four practice trials, they were still presented the test trials.

**Test Trials.** Before the start of the first test trial, the child was briefed on the entire procedure again. In cases where the child repeatedly chose the same picture twice in a row, the experimenter changed the prompt to “Choose a different picture.” If the child persisted in choosing the same picture, an even simpler prompt, “Choose a picture that is not the same,” was given. Once the prompt was changed, this formulation was retained for the rest of the test. In cases where the child playfully chose different pictures on the same stimulus card, the experimenter removed the sheet and made the child re-attend by saying, “Show me just one image” before the sheet was presented to the child again.

The task consisted on 4 practice trials containing images in arrays of 2 and 12 test trials where each trials was presented in arrays from 2 to 7, with each array size repeated twice. There was no termination rule for the ROST. Individual test trials were scored 1 (correct) or 0 (incorrect) based on whether the child re-selected the same image. The scores for individual trials were then added up to give the total score for the ROST.
